# Interferon-γ from Brain Leukocytes Enhances Meningitis by Type 4 *Streptococcus pneumoniae*

**DOI:** 10.3389/fmicb.2015.01340

**Published:** 2015-12-01

**Authors:** Elena Pettini, Fabio Fiorino, Anna Maria Cuppone, Francesco Iannelli, Donata Medaglini, Gianni Pozzi

**Affiliations:** Laboratorio di Microbiologia Molecolare e Biotecnologia, Dipartimento di Biotecnologie Mediche, Università degli Studi di SienaSiena, Italy

**Keywords:** IFN-γ, meningitis, *Streptococcus pneumoniae*, TIGR4, murine model, intracranial infection

## Abstract

*Streptococcus pneumoniae* is the leading cause of bacterial meningitis. Pneumococcal meningitis is a life-threatening disease with high rates of mortality and neurological sequelae. Immune targeting of *S. pneumoniae* is essential for clearance of infection; however, within the brain, the induced inflammatory response contributes to pathogenesis. In this study we investigate the local inflammatory response and the role of IFN-γ in a murine model of pneumococcal meningitis induced by intracranial injection of type 4 *S. pneumoniae*. Lymphoid and myeloid cell populations involved in meningitis, as well as cytokine gene expression, were investigated after infection. Animals were treated with a monoclonal antibody specific for murine IFN-γ to evaluate its role in animal survival. Intracranial inoculation of 3 × 10^4^ colony-forming units of type 4 strain TIGR4 caused 75% of mice to develop meningitis within 4 days. The amount of lymphocytes, NK cells, neutrophils, monocytes and macrophages in the brain increased 48 h post infection. IFN-γ mRNA levels were about 240-fold higher in brains of infected mice compared to controls. Pro-inflammatory cytokines such as IL-1β and TNF-α, and TLR2 were also upregulated. *In vivo* treatment with anti-IFN-γ antibody increased survival of infected mice. This study shows that IFN-γ produced during meningitis by type 4 *S. pneumoniae* enhances bacterial pathogenesis exerting a negative effect on the disease outcome.

## Introduction

Meningitis by *Streptococcus pneumoniae* (pneumococcus) is one of the most prevalent among bacterial meningitis and it is characterized by a high fatality and a high risk of neurological sequelae (Kornelisse et al., 1995; Merkelbach et al., 2000; van de Beek et al., 2002; Bogaert et al., 2004; Ramakrishnan et al., 2009; Edmond et al., 2010; Kim, 2010; Koedel et al., 2010b; Mook-Kanamori et al., 2011; Barichello et al., 2015). Neuronal injury is caused by the joint action of the direct toxicity of bacterial components and the strong inflammatory host response (Nau and Brück, 2002; Koedel et al., 2010a,b; Barichello et al., 2012). Mouse models of meningitis are used both to dissect the molecular pathogenesis of the pneumococcal infection of the brain, and to investigate novel therapeutic approaches (Chiavolini et al., 2004, 2008; Hirst et al., 2004, 2008; Banerjee et al., 2010; Woehrl et al., 2011; Mook-Kanamori et al., 2012; Tan et al., 2015). Experimental studies, aimed to develop new adjunctive therapies to be combined with antimicrobial treatment, have recently identified *in vivo* inhibition of cytokines as a promising target. During pneumococcal meningitis, bacterial components stimulate the release of inflammatory cytokines such as TNF-α, IL-1β, and IFN-γ (Wellmer et al., 2001; Zwijnenburg et al., 2003). Although the role of IFN-γ was extensively studied in viral infections, its role in acute bacterial infection is not completely understood and needs to be further investigated. IFN-γ is mainly secreted by natural killer (NK) but also by natural killer T (NKT) cells and monocytes as part of the innate immune response, and by CD4 and CD8 T lymphocytes as effector mechanism once antigen-specific immunity develops (Schoenborn and Wilson, 2007; Mildner et al., 2008). IFN-γ is an important mediator of multiple immune pathways during inflammation (Schroder et al., 2004) and was found in the cerebrospinal fluid (CSF) of patients with pneumococcal meningitis, in concentrations significantly higher than in patients with meningococcal or haemophilus meningitis (Glimåker et al., 1994; Kornelisse et al., 1997; Coutinho et al., 2013; Grandgirard et al., 2013).

The first evidence for a key role of IFN-γ in the pathogenesis of pneumococcal meningitis was obtained using a type 3 strain of *S. pneumoniae* in a mouse model of meningitis (Mitchell et al., 2012). To determine whether the observed role of IFN-γ is specific for type 3 strains or it is a general trait of pneumococcal meningitis, we used type 4 strain TIGR4, which is considered a prototype of all *S. pneumoniae* strains (Tettelin et al., 2001). In fact, type 3 differs significantly from other pneumococci in important biological traits including major virulence factors such as the polysaccharide capsule and the surface protein PspC (Sørensen et al., 1990; Janulczyk et al., 2000; Iannelli et al., 2002; Bentley et al., 2006). In this work, type 4 strain TIGR4 was used to induce meningitis in the murine model, to investigate IFN-γ gene expression, leukocyte recruitment in the brain, IFN-γ producing cells, and antibody-mediated *in vivo* neutralization of IFN-γ activity.

## Materials and methods

### Mice

Seven-weeks old female C57BL/6J, purchased from Charles River (Lecco, Italy), were maintained under specific pathogen-free conditions in the animal facilities at the University of Siena, and treated according to national guidelines (Decreto Legislativo 26/2014). All animal studies were approved by the Ethics Committee “Comitato Etico Locale dell'Azienda Ospedaliera Universitaria Senese” and the Italian Ministry of Health (authorization of the 20^th^ September, 2011).

### Bacterial strains, media, and growth conditions

*S. pneumoniae* TIGR4 (type 4) was grown in Tryptic Soy Broth (TSB, Becton Dickinson, Italy) and stored at −80°C with 10% glycerol. Solid media were prepared by addition of 1.5% agar and 3% defibrinated horse blood (Liofilchem, Italy) to TSB. Counts of colony forming units (CFU) were performed on blood-agar plates at 37°C with 5% CO_2_.

### Experimental model of meningitis and sample collection

For induction of meningitis, mice were anesthetized by intraperitoneal (i.p.) injection of xylazine hydrochloride (4 mg/Kg of mouse, Bio 98 S.r.l., Italy) and zolazepam tiletamine (15 mg/Kg of mouse, Virbac S.r.l., Italy). Animals were inoculated by the intracranial subarachnoidal route, as previously described (Chiavolini et al., 2004), using a micro-syringe with 27 gauge needles (Becton Dickinson, USA), inserting the needle between the hemispheres to a depth of 2–3 mm. Ten microliters of inoculum (3 × 10^4^ CFU/mouse) was injected into the third ventricle. Sham-infected mice received 10 μl of TSB medium. In some experiments, IFN-γ was neutralized by intracranial administration of 30 μg anti-IFN-γ (clone R4-6A2, Mabtech, Sweden) or IgG isotype control (clone eBRG1 rat IgG1 isotype control, eBioscience, USA) mixed with bacterial inoculum, in a total volume of 30 μl/mouse. Disease severity was graded using end-points on a scale of 0–5, with 0: normal; 1: piloerection and decreased spontaneous activity; 2: hunched position and loss of vigilance; 3: turns upright in >5 s when positioned on the back; 4: does not turn upright; 5: moribund. Animals were euthanized if/when they reached a score of 4. Survival was recorder for 8 days, with a daily evaluation of clinical score and the weight of individual mice.

Infected mice were transcardially perfused with ice cold PBS (Sigma-Aldrich, USA). Brains were collected from individual mice to perform phenotypic and intracellular flow cytometric analysis, to evaluate cytokine production, gene expression and bacterial load. To determine bacterial load, infected mice were aseptically transcardially perfused with ice cold PBS (Sigma-Aldrich) and half brain/mouse was plated on multilayer plates, as previously described (Iannelli and Pozzi, 2004). Plates were incubated for 48 h at 37°C with 5% CO_2_.

### Flow cytometric analysis

Brains were collected in un-supplemented RPMI 1640 (Sigma-Aldrich) and mashed onto nylon screens (Sefar Italia, Italy). For characterization of leukocyte populations by flow cytometry, cell suspension was digested with collagenase (0.5 mg/ml, Sigma-Aldrich) and DNase I (28 IU/ml, Sigma-Aldrich) for 20 min at room temperature with occasional agitation, and then triturated several times to break up tissue clumps and returned to incubate at 37°C for a further 20 min. Following enzymatic digestion, to remove low density dead cells, debris and myelin, cells were pelleted and resuspended in 7 ml of 30% (v/v) Percoll (Sigma-Aldrich) in PBS-5% (v/v) fetal calf serum (FCS, Life Technologies, USA) then centrifuged at 1400 × g for 20 min at 4°C. The upper Percoll layers were carefully removed and the cell pellet resuspended in PBS-5% (v/v) FCS. After washing cells in PBS-5% (v/v) FCS, contaminating erythrocytes were lysed by RBC lysis buffer (eBioscience), according with the manufacturer's protocol. Cell suspension was labeled with LIVE/DEAD Yellow Cell Stain Kit (Life Technologies) according to the manufacturer's instruction and incubated for 20 min at 4°C. After washes in PBS, CD16/32 mAb (clone 93, eBioscience), diluted 1:100 in PBS-3% (v/v) FCS, was added in a volume of 25 μl/sample for 20 min at 4°C, followed by incubation with a mix of fluorescent antibodies in PBS-3% (v/v) FCS in a final volume of 50 μl/sample for others 20 min at 4°C. After staining, cell suspension was fixed with fixation buffer (Becton Dickinson), according with the manufacturer's protocol.

Brain cells for intracellular cytokine assay were prepared using isolation and staining procedures, as described below. Mice received an i.p. injection of 250 μg brefeldin A ([BFA, Sigma-Aldrich], 100 μl/mouse in saline solution) 6 h before the sacrifice. Following euthanasia, performed 48 h post infection, brains were collected into ice cold RPMI 1640 containing 10 μg/ml BFA and disaggregated pressing through nylon screens of 100 μm (Becton Dickinson). Cell suspension was pelleted, resuspended in 30% Percoll containing BFA and centrifuged at 1400 × g for 20 min at 4°C. Contaminating erythrocytes were lysed as previously described. After extracellular staining performed as described above, with the addition of BFA to the mix of antibodies, the BD Cytofix/Cytoperm kit (Becton Dickinson) was used according with the manufacturer's protocol for the intracellular staining. Cellular suspension was stained with: PE-Cy7 conjugated anti-mouse CD335 (Nkp46, clone 29A1.4, eBioscience), PerCP-Cy5.5 conjugated anti-mouse Ly6G (Gr-1, clone RB6-8C5, eBioscience), APC-eFluor 780 conjugated anti-mouse F4/80 (clone BM8, eBioscience), Alexa Fluor 488 conjugated anti-mouse CD3e (clone 145-2c11, eBioscience), PE-CF594 conjugated anti-mouse CD11b (clone M1/70, Becton Dickinson), Horizon V500 conjugated anti-mouse CD45 (clone 30-F11, Becton Dickinson), Brillant Violet conjugated anti-mouse Ly6C (clone AL-21, Becton Dickinson), Alexa Fluor 700 conjugated anti-mouse CD45R (B220, clone RA3-6B2, eBioscience), APC conjugated anti-mouse IFN-γ (clone XMG1,2, eBioscience). Markers were set according to the respective IgG isotype controls. All samples were acquired on a five-laser LSR II (Becton Dickinson Biosciences), and data were analyzed using FlowJo software (Tree Star, USA).

### Quantitative RT-PCR

Brain hemispheres were collected in RNA Later (Ambion, Thermo Fisher, USA) and stored at −80°C. RNA Later solution was removed from thawed samples and 40 mg of tissue were homogenized in RA1 lysis buffer (Macherey Nagel, Germany) using 5 mm stainless steel beads (Qiagen, Germany) in Tissue Lyser (Qiagen). Total RNA was extracted with a Nucleospin RNA II isolation kit (Macherey Nagel) following the manufacturer's protocol. Integrity and quantification of RNA were evaluated using the Agilent RNA 6000 Nano Kit (Agilent Technologies, Italy) on the Agilent 2100 Bioanalyzer (Agilent Technologies). RNA samples were stored at −80°C until reverse transcription. Complementary DNA was obtained from 1 μg of total RNA for each sample using Quantitect Reverse Transcription Kit (Qiagen). Real Time PCR was performed to evaluate IFN-γ, TNF-α, IL-1β, IL-10, TGF-β, TLR2, TLR4, and TLR9 gene expression by 480 Light Cycler (Roche, Switzerland) using Sybr Green Light Cycler 480 mix (Roche) with cycling conditions as follows: 5 min 95°C denaturation and then 40 repeats of step amplification cycle (95°C for 10 s; 60°C for 10 s; 72°C for 10 s). Sequences of the primers are as follows: IFN-γ, forward, 5′-CAGCAACAGCAAGGCGAAA-3′, reverse, 5′-GCTGGATTCCGGCAACAG-3′; TNF-α, forward, 5′-CGTCGTAGCAAACCACCAAGT-3′, reverse, 5′-CCTTGAAGAGAACCTGGGAGT-3′; IL-1β, forward, 5′-GATGAAGGGCTGCTTCCAAAC-3′, reverse, 5′-ATCCCATGAGTCACAGAGG-3′; IL-10, forward, 5′-GGTTGCCAAGCCTTATCGG-3′, reverse, 5′-TCTTCACCTGCTCCACTGCC-3′; TGF-β1, forward, 5′-CGCAACAACGCCATCTATGAG-3′, reverse, 5′-GTATCAGTGGGGGTCAGCAG-3′; TLR2, forward, 5′-GTGTCTGGAGTCTGCTGTG-3′, reverse, 5′-GGAGCTTTCTTGGGCTTC-3′; TLR4, forward, 5′-CAGAGCCGTTGGTGTATC-3′, reverse, 5′-CCCATTCCAGGTAGGTGT-3′; TLR9, forward, 5′-CTGCCTTGCTCTGTCTTAC-3′, reverse, 5′-CTGTTCTGTGTGGGTCTG-3′; RPL13a, forward, 5′-CTTAGGCACTGCTCCTGTGGGT-3′, reverse, 5′-GGTGCGCTGTCAGCTCTCTAAT-3′.

RPL13a encoding gene was utilized as endogenous gene. The relative gene expression levels in each sample were normalized against the brains of uninfected mice using the ΔΔCt method (Livak and Schmittgen, 2001).

### Statistical analysis

Survival experiments were analyzed by the Kaplan-Meier method, using the Log-Rank test. Statistical significance among two independent groups (i.e., infected and control groups) was determined by two tailed Mann-Whitney test. Statistical significance was defined as *P* ≤ *0.05*. Graphpad 4.0 software was used for analysis.

## Results

### Analysis of intracranial infection by type 4 *S. pneumoniae*

Pneumococcal meningitis was induced in inbred C57BL/6J mice inoculating the wild type *S. pneumoniae* serotype 4 (TIGR4) by the intracranial subarachnoidal route as previously described (Chiavolini et al., 2004). Survival and clinical parameters were daily evaluated up to 8 days post infection.

In order to identify the minimum amount of bacteria required for inducing meningitis, groups of 4 mice were infected by the intracranial route with four scalar doses of TIGR4, between 3 × 10^3^ and 1 × 10^5^ CFU/mouse. The dose of 3 × 10^4^ CFU/mouse killed 50 and 75% of mice 3 and 4 days post infection, respectively. Similar percentages were observed in mice infected with 10^5^ CFU/mouse, while both lower doses of 3 × 10^3^ and 10^4^ CFU had higher survival percentages (data not shown).

**Figure 1:**
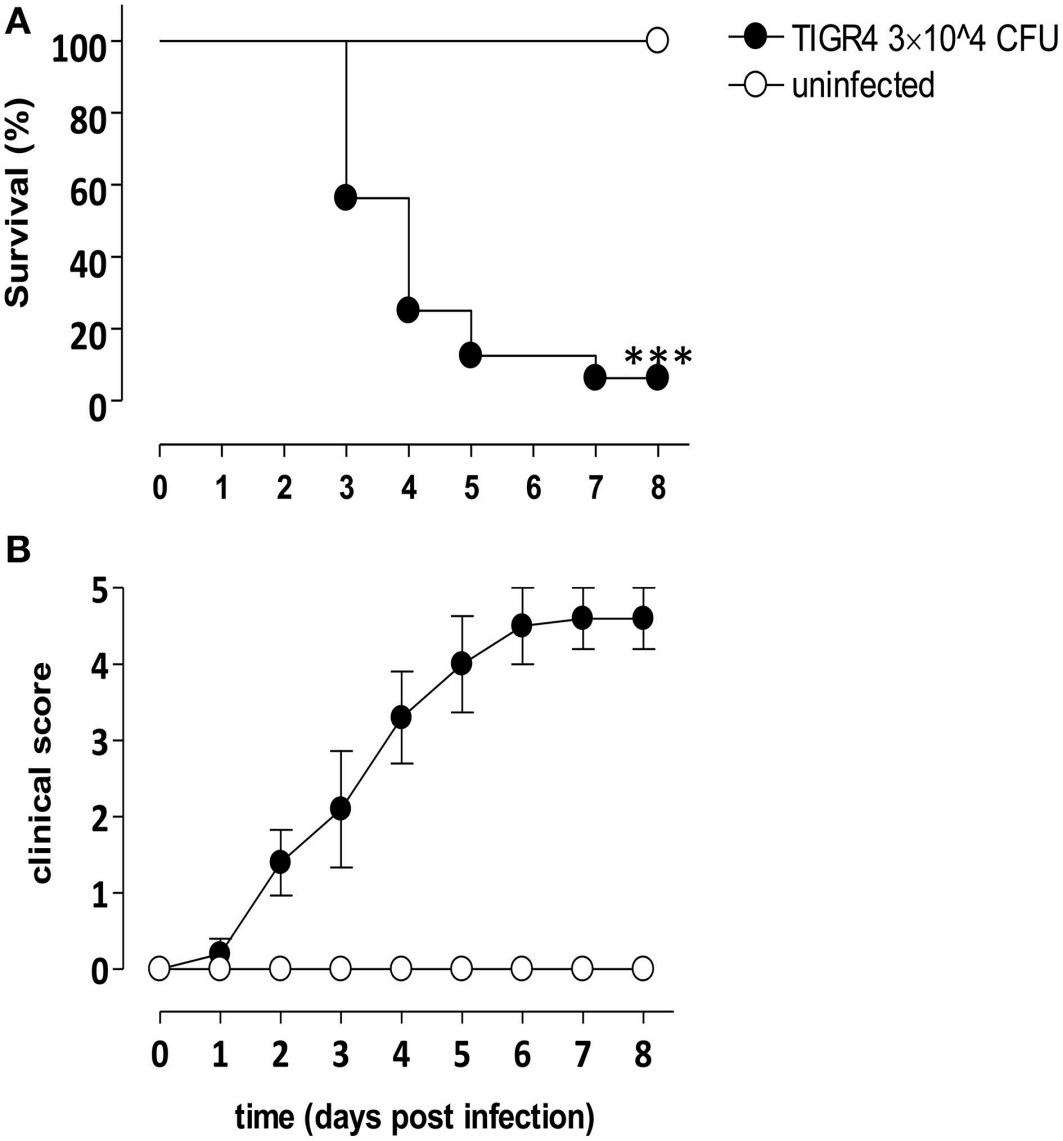
**Murine model of meningitis by *S. pneumoniae*.** C57BL/6J mice were infected by the intracranial route with 3 × 10^4^ CFU of *S. pneumoniae* strain TIGR4 (filled circles) or injected with bacterial growth medium (control, open circles). Data are from two independent experiments with 8 mice/group, and animals were monitored up to 8 days post infection. **(A)** Kaplan-Meier curve of mouse survival. Results were expressed as percentage of survival over time, and statistical differences were evaluated between infected mice and control group using Log-Rank test. Asterisks indicate statistical significance (^***^P < 0.0001). **(B)** Clinical score analysis was evaluated using a scale for severity of disease, from 0 (normal) to 5 (moribund), and values were reported as mean value ± SEM.

The amount of type 4 *S. pneumoniae* chosen for intracranial challenge experiments was 3 × 10^4^ CFU/mouse. Groups of 8 mice were infected with *S. pneumoniae* TIGR4 or injected with bacterial growth medium. Three days post infection with TIGR4, 56% of infected mice survived and values decreased until 8 days post infection (Figure 1A), when about 6% of mice survived. Median survival time of mice challenged with TIGR4 was 96 h. On the contrary, all mice of the control group, injected by the intracranial route with the bacterial growth medium, survived with no signs of disease. Survival analysis was supported by daily clinical parameters observation, in terms of weight and clinical score. A decrease of about 25% of initial weight was estimated starting from 3 days post infection (data not shown). The clinical score of infected mice gradually increased following infection, while all control mice showed no signs of illness and no differences in clinical score compared to initial condition (Figure 1B).

Our data, analysing survival and clinical parameters in a murine model of intracranial infection with *S. pneumoniae* TIGR4, suggested that the dose of 3 × 10^4^ CFU/mouse and the time point of 48 h from challenge are optimal for the subsequent characterization of the induced immune reaction since mice survived but signs of the meningitis were evident.

### Gene expression in brain of mice with meningitis

**Figure 2:**
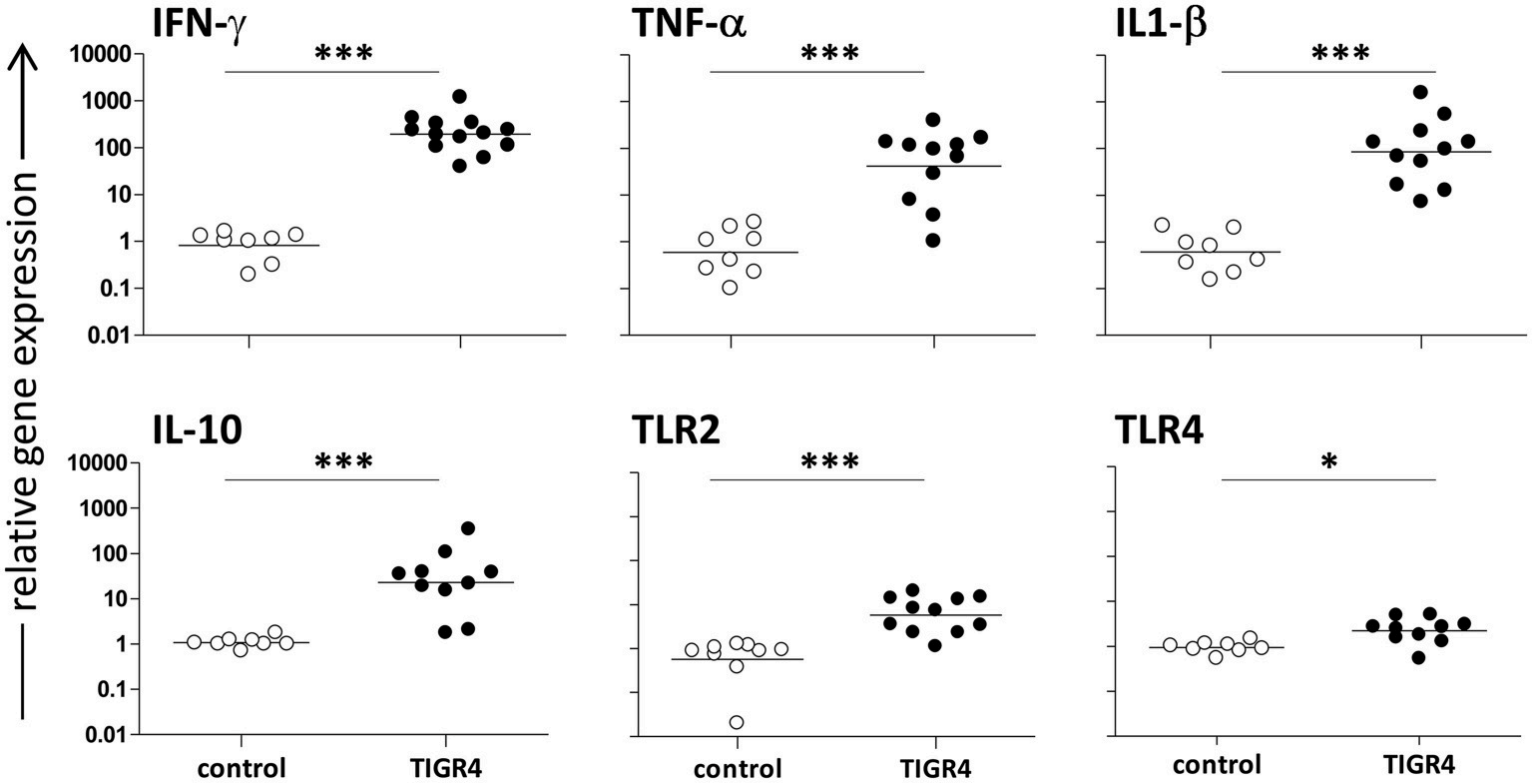
**Cytokine and TLR gene expression in the mouse brain 48 h post infection**. The relative IFN-γ, TNF-α, IL-1β, IL-10, TLR2, and TLR4 gene expression was determined by RT-PCR in brains of C57BL/6J mice infected by the intracranial route with 3 × 10^4^ CFU of *S. pneumoniae* TIGR4 (filled circles). As control, a group of mice was injected with bacteria growth medium (open circles). All animals were sacrificed 48 h post infection. Symbols represent individual mice and bars represent the geometric mean of each group. Data are from two independent experiments with 4–6 mice/group. Statistical analysis was performed on log-transformed data between infected and control group using the two tailed Mann-Whitney test. Asterisks indicate statistical significance (^*^P ≤ *0.05*; ^***^P ≤ *0.001*).

Mice challenged with *S. pneumoniae* TIGR4 were sacrificed 48 h post infection (*n* = 4–6 mice/group in two independent experiments). Brains were collected and mRNAs were extracted in order to evaluate gene expression of cytokines and TLRs during meningitis (Figure 2). Interestingly, intracranial infection with 3 × 10^4^ CFU/mouse of TIGR4 induced a significant IFN-γ gene expression with an increase of about 240-folds compared to the control group injected with bacterial growth medium (Figure 2; *P* < 0.001). Also the gene expression of inflammatory cytokines such as TNF-α and IL-1β in brains of infected mice showed a considerable relative increase of about 70- and 140-folds, respectively, compared to the control group (*P* < 0.001, Figure 2). Gene expression of the regulatory cytokine IL-10 increased of about 20-folds (*P* < 0.001, Figure 2). On the contrary, no differences between infected and control mice was observed for TGF-β gene expression at this time point (data not shown). The analysis of TLR gene expression during *S. pneumoniae* meningitis showed a significant increase of about 10-folds (*P* < 0.001) and 3-folds (*P* < 0.05) for TLR2 and TLR4, respectively (Figure 2), while no statistically significant increase was observed for TLR9 (data not shown).

### Leukocyte recruitment and source of IFN-γ in brain of mice with *S. pneumoniae* meningitis

**Figure 3:**
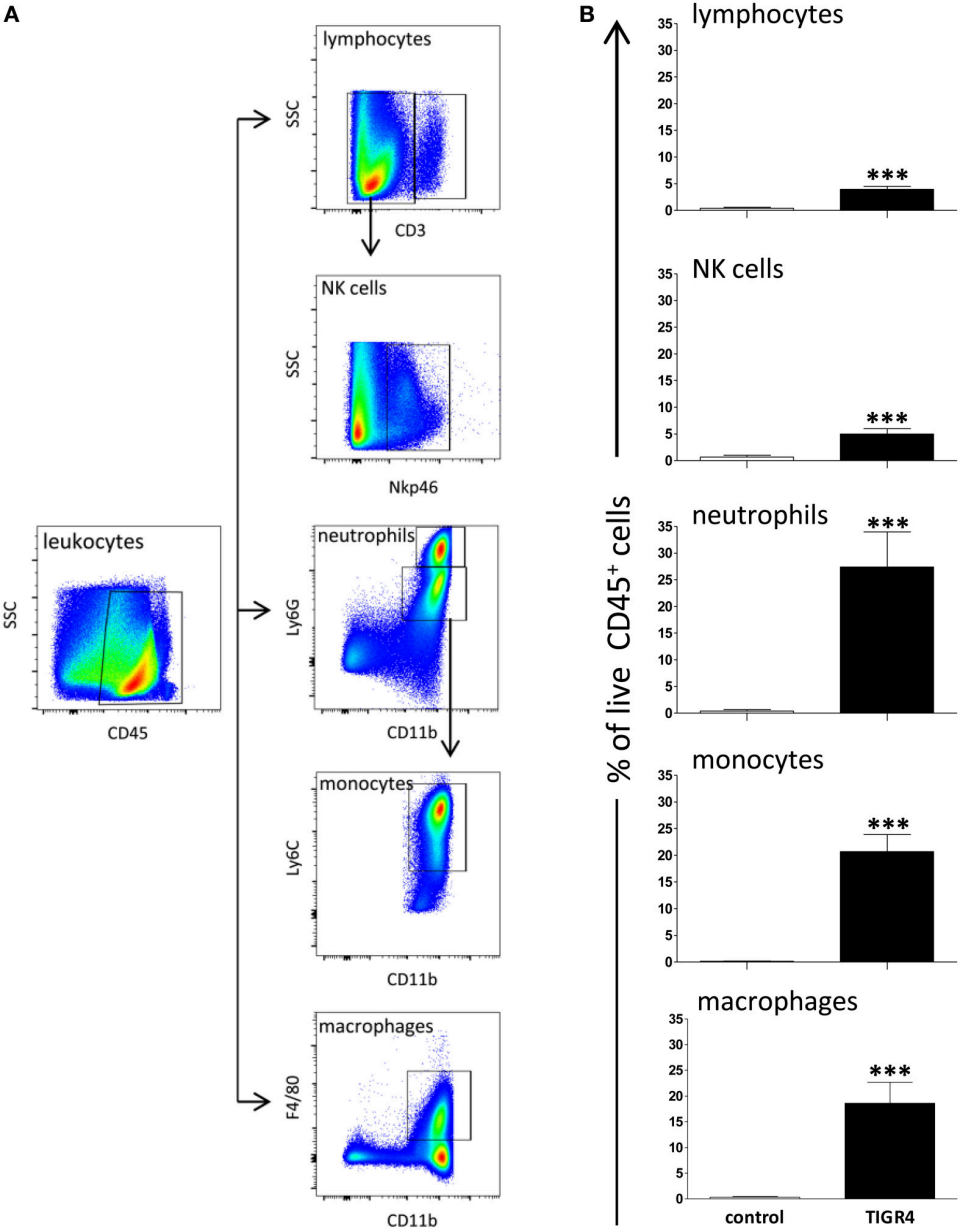
**Leukocytes in the brain 48 h post infection**. The recruitment of leukocytes was quantified by multiparametric flow cytometric analysis in the brains of C57BL/6J mice collected 48 h post intracranial infection with 3 × 10^4^ CFU of *S. pneumoniae* TIGR4. As control, a group of mice was injected with bacterial growth medium. **(A)** Gating strategy adopted for the identification of leukocyte populations in brain of mice. Lymphocytes (CD45^hi^CD3^+^), NKs cells (CD45^hi^CD3^−^NKp46^+^), neutrophils (CD45^hi^CD11b^hi^Ly6G^+^), monocytes (CD45^hi^CD11b^hi^Ly6G^−^Ly6C^+^), and macrophages (CD45^hi^CD11b^hi^F4/80^+^) were detected. **(B)** Percentages (%) of cell populations detected in brain of mice infected with *S. pneumoniae* TIGR4 or injected with bacterial growth medium. Infection with TIGR4 induced the entry in the brain of about 4% lymphocytes, 5% NK cells, 27% neutrophils, 21% monocytes, and 19% macrophages, all evaluated on CD45^+^ cells. In control group all cell populations detected were ≤ 0.7% of CD45^+^ cells. Data are from four independent experiments with 3–6 animals/group. Bars represent mean ± SEM. Two tailed Mann-Whitney test was used for comparison between infected and control group and asterisks indicate statistical significance (^***^P < 0.001).

**Figure 4:**
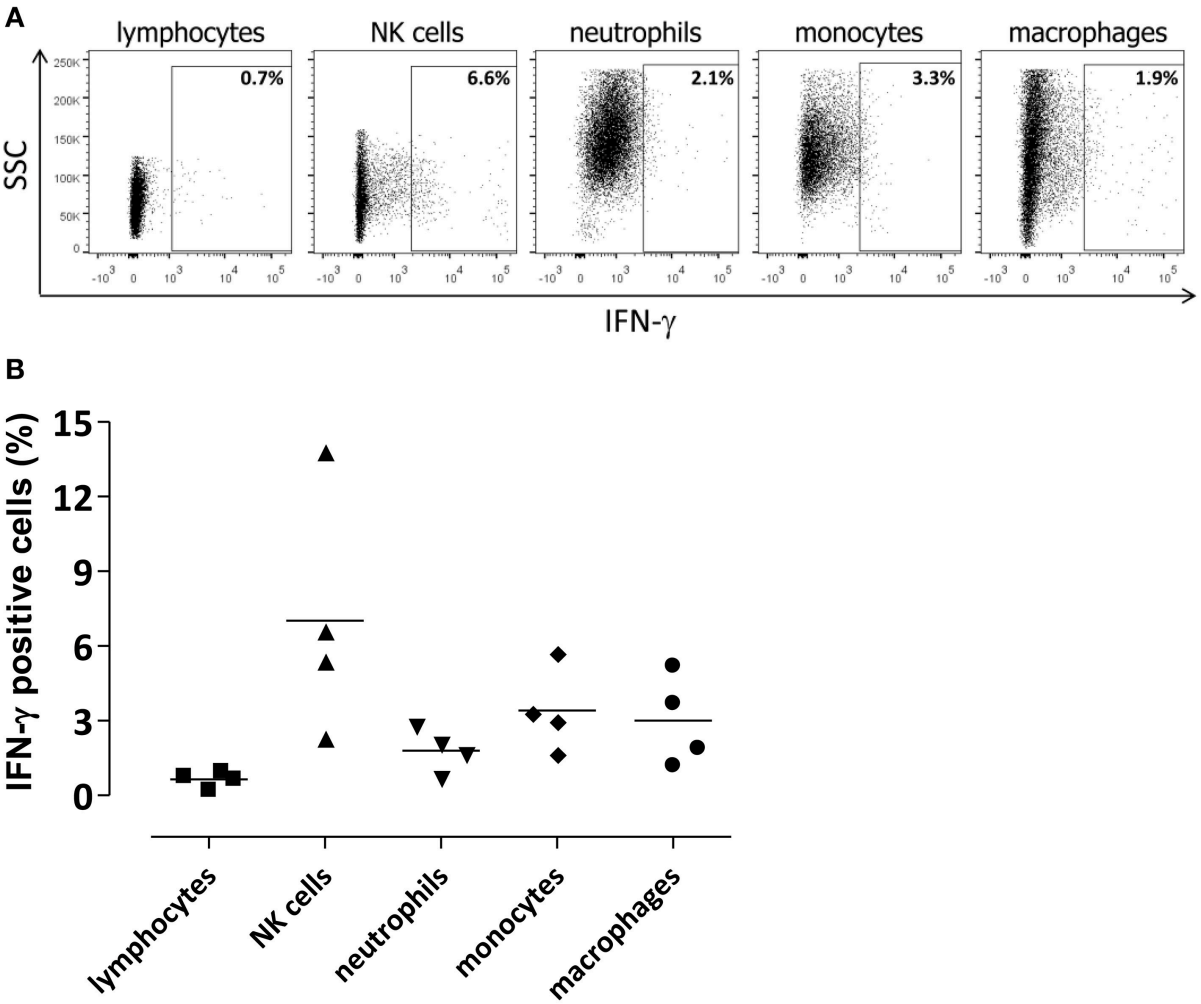
**Cellular source of IFN-γ in the infected brain**. Lymphocytes (CD45^hi^CD3^+^), NKs (CD45^hi^CD3^−^NKp46^+^), neutrophils (CD45^hi^CD11b^hi^Ly6G^+^), monocytes (CD45^hi^CD11b^hi^ Ly6G^−^Ly6C^+^), and macrophages (CD45^hi^CD11b^hi^F4/80^+^) were analyzed for IFN-γ intracellular staining. Flow cytometric analysis was performed in brain of mice 48 h post intracranial infection with 3 × 10^4^ CFU of *S. pneumoniae* TIGR4. **(A)** Representative flow cytometry dot plot graphs showing the percentage of IFN-γ^+^ cells among different cell populations in the brain. The gate for IFN-γ^+^ cells was set using an appropriate APC-conjugated isotype control. **(B)** Symbols represent individual mice and bars represent the mean. Mean values for each cell population were 0.6 ± 0.1% for lymphocytes, 7 ± 2.4% for NK cells, 1.8 ± 0.4% for neutrophils, 3.4 ± 0.8% for monocytes, and 3 ± 0.9% for macrophages.

To characterize pneumococcal meningitis, leukocyte recruitment was analyzed in brains of mice with meningitis 48 h post infection with TIGR4 (*n* = 3–6 mice/group in four independent experiments) (Figures 3A,B) and the source of IFN-γ was investigated by flow cytometric analysis (Figure 4). The average number of leukocytes (CD45^+^ live cells) detected in mice was about 6.4 × 10^4^/brain. Neutrophils (CD45^hi^CD11b^hi^Ly6G^+^ cells) were the most abundant cell population in the inflammatory infiltrate detected in TIGR4 infected brains, representing the 27.4 ± 6.5% (mean ± SEM) of CD45^+^ cells (*P* < 0.001 vs. control, Figure 3B). Within the leukocyte population detected in the brain of TIGR4 infected mice, was also observed a significant increase of monocytes (CD45^hi^CD11b^hi^Ly6G^−^Ly6C^+^ cells) representing the 20.7 ± 3.1% of CD45^+^ cells (*P* < 0.001), macrophages (CD45^hi^CD11b^hi^F4/80^+^ cells) with the 18.6 ± 4% (*P* < 0.001), lymphocytes (CD45^hi^CD3^+^ cells) with the 3.9 ± 0.5% (*P* < 0.001) and NKs (CD45^hi^CD3^−^NKp46^+^ cells) with the 5 ± 1% (*P* < 0.001) (Figure 3B). B cells (CD45^hi^B220^+^ cells) were also investigated and corresponded to 0.3 ± 0.1% of leukocytes (data not shown). In the brain of control mice, injected with bacterial growth medium, only few leukocytes were observed with 0.4 ± 0.3% of neutrophils, 0.07 ± 0.04% of monocytes, 0.3 ± 0.1% of macrophages, 0.4 ± 0.15% of lymphocytes and 0.7 ± 0.15% of NK cells.

To characterize the IFN-γ involvement during pneumococcal meningitis, cells expressing IFN-γ were analyzed in the brain by intracellular staining (Figures 4A,B). Up to 14% of NKs were positive for IFN-γ staining 48 h post infection. Not surprisingly, also monocytes, macrophages, and neutrophils produced lower amounts of IFN-γ (about 3%; Figure 4B). The MFI of NKs producing IFN-γ was about 10 times higher than the MFI of the other cell populations analyzed, confirming that NKs are the main source of IFN-γ in brain of *S. pneumoniae* TIGR4 infected mice. No IFN-γ staining was detected in control mice injected by the intracranial route with bacterial growth medium (data not shown).

### IFN-γ antibody-mediated neutralization

**Figure 5:**
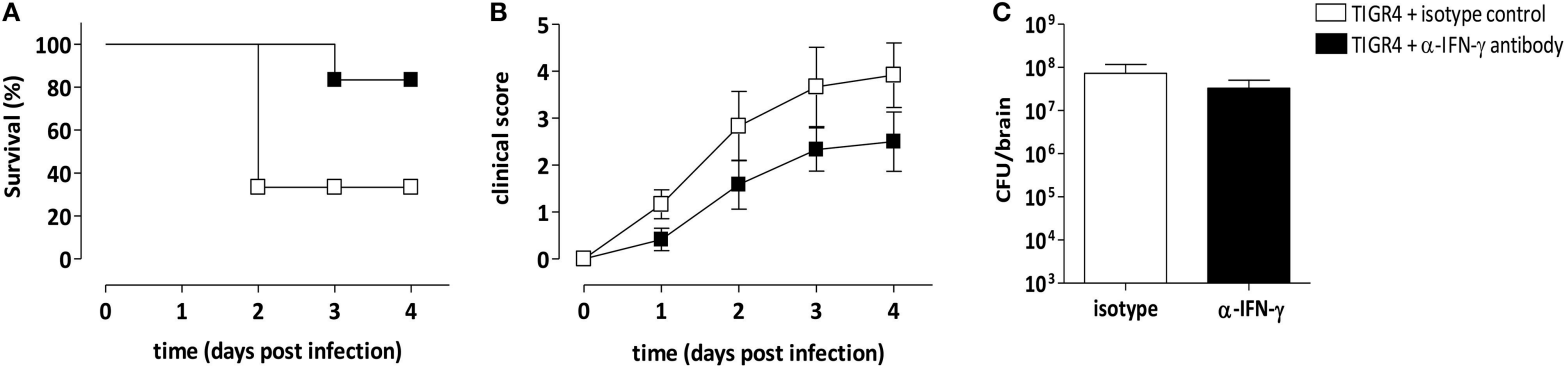
**IFN-γ specific monoclonal antibody treatment**. Mice were infected by the intracranial route with 3 × 10^4^ CFU of *S. pneumoniae* TIGR4 and administered with a neutralizing anti-IFN-γ antibody (black) or with an isotype control antibody (white) at the time of the bacteria inoculum. To analyze the role of IFN-γ in pneumococcal meningitis, animals were monitored up to 4 days post infection. Data are from two independent experiments with 4 mice/group. **(A)** Kaplan-Meier curve of mouse survival. Results were expressed as percentage of survival over time. **(B)** Clinical score was evaluated using a scale for severity of disease, from 0 (normal) to 5 (moribund), and reported as mean value ± SEM. **(C)** Bacterial load estimation in brain of mice 48 h post infection. Mean values ± SEM are reported.

The involvement of IFN-γ produced in the brain during meningitis with *S. pneumoniae* TIGR4 was further confirmed by IFN-γ antibody-mediated neutralization. Anti-IFN-γ antibody was administered by the intracranial route at the time of infection and survival was analyzed. As control, a group of mice was infected with bacteria and administered with the isotype control. Two independent experiments were performed, each with 4 mice/group. Two days post infection all mice infected with TIGR4 and treated with the anti-IFN-γ antibody survived, whereas only the 33% of animals treated with the isotype control survived (*P* = 0.019, Figure 5A). Eighty-three percent of mice treated with the IFN-γ neutralizing antibody survived 3 and 4 days post infection, while the survival rates of isotype control treated mice didn't change, with 33% of survived animals (Figure 5A). Differences of mice survival between the two groups were reduced at day 8, where 33% of mice treated with anti-IFN-γ antibody and 17% of mice in the control group survived, respectively (data not shown). All infected mice were monitored for clinical signs (Figure 5B) up to 4 days post infection. Interestingly, the inoculum of anti-IFN-γ antibody caused minor clinical symptoms, compared to control group, supporting the survival data collected at this early time point.

Bacterial load was also estimated in infected brains 48 h post infection and data demonstrated that the intracranial administration of anti-IFN-γ antibody didn't influence the presence of bacteria compared to isotype control treated mice, with values of about 10^8^ CFU/brain (Figure 5C).

## Discussion

In this work, we demonstrate the key role of IFN-γ in driving the pathogenesis of meningitis by serotype 4 *S. pneumoniae*. In particular, using the murine model of pneumococcal TIGR4 meningitis, we have shown: (i) high expression of IFN-γ in the brain of infected mice, (ii) recruitment in the brain of IFN-γ-producing leukocytes, (iii) protection of mice from lethality by the *in vivo* IFN-γ antibody-mediated neutralization.

It is known that pneumococcus infection and the host's consequent inflammatory reactions are evident causes of clinical deterioration and neurologic symptoms, but the understanding of the interaction between bacterial products and the host's inflammatory response leading to neuronal damage is still incomplete (Gerber et al., 2004). IFN-γ is a key factor in brain during meningitis by *S. pneumoniae*, it activates the immune response but it also may enhance the pathology (Schroder et al., 2004). It regulates chemokines and adhesion molecules involved in the trafficking of leukocyte to the site of inflammation (Schroder et al., 2004) and modulates a range of processes involved in host immunity in response to *S. pneumoniae* such as enhancing the function of macrophages and polymorphonuclear leukocytes and the release of inflammatory mediators (Gil et al., 2001). It was recently shown that IFN-γ is a major driver of pathology during pneumococcal meningitis using serotype 3 *S. pneumoniae* (Mitchell et al., 2012). The type 3 strain constitutes a peculiar group among pneumococci and it distinguishes from other serotypes mainly for the polysaccharide capsule, not covalently attached to the cell wall (Sørensen et al., 1990; Bentley et al., 2006), and the surface protein PspC (Janulczyk et al., 2000; Iannelli et al., 2002). Here, we characterized the role of IFN-γ in acute meningitis induced by serotype 4 *S. pneumoniae*. To this aim we have adopted the murine model of pneumococcal meningitis, based on the inoculation of bacteria into the subarachnoid space (Chiavolini et al., 2004) and we have adapted it to C57BL/6 mice. This method assures the development of the pathology and may be particularly useful to investigate host-pathogen interactions and to develop novel therapeutic approaches.

The considerable involvement of IFN-γ during meningitis by TIGR4 *S. pneumoniae* was demonstrated by the robust increase of about 240-folds of mRNA gene expression in brains of infected mice compared to uninfected animals. This is in line with data reported in meningitis induced by the serotype 3 *S. pneumoniae* (Mitchell et al., 2012). In our work, the role of IFN-γ in driving the pathology during type 4 *S. pneumoniae* meningitis was further confirmed *in vivo* by the IFN-γ antibody-mediated neutralization. The survival rate was significantly higher at early time points after challenge in mice treated with the IFN-γ blocking antibody compared to the animals treated with the isotype control. These data were supported also by the reduction of clinical score values. Interestingly, IFN-γ neutralization did not significantly affect the amount of bacteria and leukocytes in brains.

The intracranial infection with TIGR4 induced the recruitment of different leukocyte populations in the brain with a high afflux of myeloid cells, mainly neutrophils, monocytes and macrophages, and also the involvement of small amounts of lymphocytes and NK cells. The pivotal role of neutrophils in acute bacterial meningitis has been previously described (Mildner et al., 2008; Mook-Kanamori et al., 2011). NK cells were previously indicated as the dominant source of IFN-γ in meningitis induced by the type 3 *S. pneumoniae* and their role in IFN-γ production was confirmed by experiments in which the depletion of NK cells greatly reduced the amount of IFN-γ in terms of both protein and mRNA within the brain (Mitchell et al., 2012). Our data indicated that also 48 h after intracranial infection with the serotype 4 TIGR4, IFN-γ is produced mainly by NKs recruited in brain. Not surprisingly, also monocytes, macrophages, and neutrophils were found to produce IFN-γ, in line with previous *in vitro* and *in vivo* studies (Munder et al., 1998; Olliver et al., 2011; Gomez et al., 2015).

We have also demonstrated the production of other inflammatory cytokines in the brain during meningitis by TIGR4, as indicated by the TNF-α and IL-1β relative gene expression increase. TNF-α and IL-1β were previously demonstrated to be involved in hippocampal damage in animal models (Bogdan et al., 1997; Leib et al., 2001). These inflammatory cytokines were also detected in CSF of pneumococcal meningitis patients with a fatal outcome (Ohga et al., 1994; Grandgirard et al., 2013), triggering damages at brain level and inducing a negative effect on the disease outcome (Nau and Brück, 2002).

Our data demonstrate the role of IFN-γ in the pathogenesis of meningitis induced by the type 4 *S. pneumonia* and further support that the down-modulation of inflammatory cytokines, mainly IFN-γ, can be considered a valid strategy for new adjunctive therapies. Optimal level of cytokines necessary to not exacerbate the host immune response and also eliminate the pathogen still needs to be deeply investigated with *in vivo* studies (Lee et al., 2007; van de Beek et al., 2012; Barichello et al., 2015).

Besides proinflammatory cytokines, our study showed that also the regulatory cytokine IL-10 gene expression increased in brain of infected mice, while no differences were observed for TGF-β. TLRs, highly involved in the initial sensing of pneumococci in the central nervous system, were analyzed by mRNA gene expression during serotype 4 *S. pneumoniae* meningitis and results showed a significant increase mainly for TLR2, but not for TLR9. The important involvement of TLR2 in pneumococcal meningitis was previously demonstrated in TLR2-deficient mice infected with *S. pneumoniae* serotype 3, showing an increase in the bacterial load and blood-brain barrier disruption (Echchannaoui et al., 2002; Koedel et al., 2003).

In conclusion, in this work we show that IFN-γ, highly expressed by different leukocyte populations in the brain of TIGR4 infected mice, enhances the development of brain injury in bacterial meningitis by serotype 4 *S. pneumoniae*. Our data highlight the negative effect of IFN-γ on the disease outcome in the murine model of type 4 pneumococcal meningitis, and its possible role as critical target in adjunctive therapies.

### Conflict of interest statement

The authors declare that the research was conducted in the absence of any commercial or financial relationships that could be construed as a potential conflict of interest.
